# A randomized and prospective study on the value of antibiotic prophylaxis administration in transurethral resection of the prostate

**DOI:** 10.1590/S1516-31802004000100002

**Published:** 2004-01-08

**Authors:** Rodrigues Paulo, Hering Flávio, Meller Alex, Campagnari João Carlos, Márcio D'Império

**Keywords:** Prostatism, Antibiotics, Prostate, Infection, Prostatismo, Antibióticos, Próstata, Infecção, Prostatite

## Abstract

**CONTEXT:**

Antibiotic prophylaxis in transurethral resection of the prostate is a regular practice in urology. However, its prophylactic effect can be questioned when the antiseptic surgical technique is used. Nonetheless, urine culture-oriented antibiotic therapy is the gold standard for avoiding improper medication usage and bacterial resistance.

**OBJECTIVE:**

To study the efficacy of antibiotic usage in patients with negative urine cultures, who were submitted to transurethral resection of the prostate.

**TYPE OF STUDY:**

Prospective open labeled study.

**SETTING:**

Tertiary care referral hospital

**PARTICIPANTS:**

124 consecutive patients, who were randomly divided into two groups to receive antibiotic prophylaxis or not.

**MAIN MEASUREMENTS:**

Cultures from meatus, urine, irrigation and antiseptic fluid, and prostate tissue chips, were compared and analyzed for bacterial sensitivity to the antibiotic used, according to the surgeon's personal criteria. McLennan's test was used for statistical analysis.

**RESULTS:**

No statistically significant difference regarding clinical evolution was found between the groups that received or antibiotics or not. Statistical significance was found regarding the occurrence of positive urine cultures during the postoperative period for those not receiving antibiotics, but not in relation to fever, prostate chip culture or bacteremic episodes.

Sixty-eight subjects (57.1%) presented positive prostatic tissue culture. There was no specific correlation between the recovered bacteria from the meatus, prostatic tissue chip and urine and the spectrum of the administered antibiotic. Six cases showed the same bacteria in the urine and prostatic tissue chip. Only fifteen cases (25%) in the antibiotic group showed the desired sensitivity directed to the collected bacteria.

**CONCLUSIONS:**

Antibiotic prophylaxis for patients whose urine is sterile is debatable in patients who are candidates for transurethral resection of the prostate. Most of the time, the antibiotic agent used is not specific for any of the bacteria recovered from the various sources analyzed.

## RESUMO

**CONTEXTO:**

A profilaxia antibiótica nas ressecções transuretrais da próstata é uma prática regular e freqüente na clínica urológica. No entanto, seu efeito profilático e bactericida protetor pode ser contestado se procedimentos assépticos são utilizados na realização da cirurgia, sobretudo em pacientes com urina estéril. No caso de infecção urinária, a identificação dos germes para escolha do antibiótico adequado pode ser necessária.

**OBJETIVO:**

Verificar a eficácia da antibioticoprofilaxia em pacientes com urina estéril submetidos a ressecção transuretral de próstata.

**TIPO DE ESTUDO:**

Prospectivo num centro de referência de tratamento urológico, aberto.

**LOCAL:**

Hospital de referência terciária.

**PARTICIPANTES:**

124 pacientes.

**VARIÁVEIS ESTUDADAS:**

124 pacientes consecutivos foram randomicamente divididos em dois grupos para receber ou não antibioticoterapia profilática na ressecção transuretral de próstata. Cultura do meato uretral, urina, líquido irrigante e anti-séptico, além dos fragmentos de próstata ressecados foram analisados quanto a sensibilidade a antibióticos, escolhidos a critério do cirurgião, e determinada a partir de antibiograma com as cepas bacterianas identificadas nos sítios mencionados.

**RESULTADOS:**

Não se encontrou diferença estatisticamente significante na evolução clínica de ambos os grupos. Aqueles que receberam antibioticoprofilaxia apresentaram menor freqüência de cultura urinária positiva do que aqueles que não receberam profilaxia. No entanto, na observação da evolução clínica de ambos os grupos, o uso de antibiótico não mostrou qualquer benefício no que concerne à ocorrência de febre, positividade das culturas obtidas dos fragmentos de próstata ressecados ou episódios de bacteremia. 68 casos (57,1%) apresentaram cultura positiva do tecido prostático. Entretanto, não houve correlação entre a bactéria identificada a partir do tecido prostático e de outros locais, tais como meato, urina, líquido irrigante ou antiséptico utilizado. Somente em seis casos foi encontrada a mesma bactéria no tecido prostático e na urina pós-operatória. Apenas em 15 casos (25%) do grupo antibiótico observou-se a sensibilidade esperada da bactéria identificada ao antibiótico utilizado.

**CONCLUSÃO:**

A profilaxia antibiótica em pacientes com urina estéril submetidos a ressecção transuretral de próstata poderia ser reavaliada, pois, na grande maioria das vezes, o antibiótico selecionado com finalidade profilática não apresenta especificadade às bactérias identificadas nos diversos sítios pesquisados.

## INTRODUCTION

Antibiotic prophylaxis is a well-known practice in urological surgery because urological instruments may introduce microorganisms into a sterile urinary tract. This longstanding practice has derived from poor understanding of bladder colonization introduced by invasive surgical methods such as cystoscopy. The physiopathology of bladder colonization has recently become better understood and it seems that the presence of infravesical obstruction is a fundamental factor in persistent colonization of the bladder chamber.^1^

Antibiotic administration has been used in transurethral resection of the prostate, even in patients presenting sterile urine preoperatively, due to awareness of the possibility of bacteremia related to urethral manipulation.^2-4^

However, some studies have claimed that bacteremic episodes are short-lived and uncontrollable, even in the presence of antibiotic coverage, especially when it is argued that fevers are related to non-bacteremic factors such as tumoral necrosis factor^5^ and not to the bacterial dissemination itself.

The objective of the present study was to make a prospective clinical evaluation of patients submitted to transurethral resection of the prostate, according to whether or not antibiotic prophylaxis was administered.

## METHODS

Among the consecutive candidates referred to our service for transurethral resection of the prostate, 124 patients agreed to participate in the study and were randomly enrolled to receive antibiotic prophylaxis or not, in an open labeled study consisting of two groups of subjects. Sixty-two patients received antibiotic (average age of 66.3 ± 14.9 years old) while the others did not receive any placebo pill (average age of 76 ± 11.1). None of the patients was previously catheterized during the preoperative period. Neurological disease, neoplasia and the detection of urinary tract infection by urine culturing were criteria for exclusion.

The antibiotic administered was chosen according to the surgeon's criteria, based on personal preference and daily practice. At the end of the study, two cases in the antibiotic group and three cases in the non-antibiotic group were discarded due to incomplete information.

The two groups established were comparable in relation to age (76 ± 11.1 years in the prophylactic group; 66.3 ± 14.9 years in the control group), quantity of resected prostatic tissue (18.1 ± 5.2 g for prophylaxis; 14.7 ± 6.3 g for control), length of the operation (98 ± 43 min for prophylaxis; 103 ± 37 min for control), use of the Foley catheter (22 or 24 Fr) and also the length of postoperative catheterization (3.3 ± 0.32 days for prophylaxis; 3.1 ± 0.49 days for control).

All the patients had their urine confirmed as sterile via culturing and none of them received any antibiotic drug during the two-week period before the operation. The perfusion fluid used was 3% mannitol.

Two fragments of the resected gland were immediately sent for culturing at the end of the operation, as well as samples of the irrigating and antiseptic fluid glutaraldehyde (Cidex®). The remaining resected prostatic fragments were histologically analyzed via hematoxylin-eosin staining.

Temperatures higher than 37.5°C and chills were considered to be bacteremic episodes.

From the second postoperative day onwards, culturing of urine from the catheter was undertaken daily, until the catheter was taken out. Swab samples from the meatal region were also obtained for culturing. Significant bacteriuria was defined as 105 or more colony-forming units/ml. If irrigation was still being provided on the second day, it was closed for 30 minutes before the urine sample for culturing was taken. Possible blood clots were relieved by aspiration.

The antibiotics utilized were intravenous (IV) gentamicin 500 mg 8/8h in 6 cases; oral cephalothin 500 mg 6/6h in 15 cases; oral trimethoprim/sulfamethoxazole 200/400 mg 12/12h in 6 cases; oral norfloxacin 400 mg 12/12h in 26 cases; IV amikacin 500 mg 12/ 12h in 2 cases; and IV ceftriaxone 1 g 24/24h in 7 cases.

Statistical analysis was performed using McLennan's test with a 95% confidence interval.

## RESULTS

No complications related to hypotension, septicemia, hemorrhage or hyponatremia were observed in the 124 cases.

Microorganisms were recovered from the prostatic tissue in 52 men (43.7% of the total sample) (Table 1), but the prevalence was the same in both antibiotic and control groups (p > 0.05) (Table 2). The whole study yielded 1,170 cultures, with 16 different microorganisms isolated but no preponderance of any (Table 1). However, the postoperative urine cultures showed markedly higher incidence of microorganisms among those that did not receive prophylaxis (p < 0.05) (Table 3). The occurrence of chills, fever and hypotension was the same in both groups (p > 0.05).

**Table 1:** Type and source of the organism isolated in 119 patients submitted to transurethral resection of the prostate with or without antibiotic prophylaxis

	Prostate		Antiseptic		Irrigating fluid		Meatus		Urine	
	C	A	C	A	C	A	C	A	C	A
*M. morgani*	4	4	1	-	-	-	-	2	-	-
*E. faecalis*	4	4	1	-	-	-	1	3	-	-
*S. epidermidis*	4	2	-	-	-	-	22	9	4	-
*E. coli*	3	3	-	-	-	-	4	6	5	3
*Enterobacter sp.*	9	4	-	-	-	-	3	3	2	2
*Pseudomonas aeruginosa*	1	1	1	-	-	-	1	1	1	2
*Citrobacter sp.*	-	3	-	-	-	-	1	-	-	1
*P. mirabilis*	2	1	-	-	-	-	1	1	-	-
*S. aureus*	-	1	-	-	-	-	6	4	1	2
*S. viridans*	-	-	-	-	-	-	1	2	-	1
*Act. calcareus*	-	-	-	-	-	-	-	1	-	-
*Klebsiella pneumoniae*	-	1	-	-	-	-	1	3	-	3
*H. influenza*	-	-	-	-	-	-	-	1	-	-
*Providencia sp.*	-	1	-	-	-	-	-	1	-	1
*S. marcencens*	-	-	-	-	-	-	1	1	-	2
*Candida sp.*	-	-	-	-	-	-	-	1	-	-
**Total**	**27**	**25**	**3**	**0**	**0**	**0**	**42**	**39**	**13**	**17**

* *Most of the cases had more than one organism isolated from different samples. The irrigating fluid utilized did not show any bacterial growth either in the antibiotic or control group. C = control; A = antibiotic group.*

**Table 2:** Culture from the prostatic resected tissue in 59 control patients and 60 antibiotic-prophylaxis patients

Tissue culture	Antibiotic prophylaxis	
	Yes	No
Positive	21	33
Negative	38	27
**Total**	**59**	**60**

*Chi-squared = 0.35; p = 0.5529; odds ratio = 0.8684; confidence interval (95%) = [0.5278; 1.4220].*

**Table 3:** Postoperative urine culture results in the 60 control group patients and 59 patients receiving prophylaxis

Urine culture	Antibiotic prophylaxis	
	Yes	No
Positive	7	24
Negative	52	36
**Total**	**59**	**60**

*Chi-squared = 10.32; p = 0.0013; odds ratio = 0.4615; confidence interval (95%) = [0.2720; 0.7622].*

The antibiotic administered showed specificity in fifteen cases: ceftriaxone in five cases (prostatic culture: four cases of *E. coli*; meatal culture: one case of *E. faecalis*); amikacin in two cases (prostatic culture: *E. coli* and *M. morgani*), norfloxacin in six cases (prostatic culture: one case of *E. coli*; urine culture: two cases of *S. epidermidis*; meatal culture: two cases of *S. epidermidis* and one of *E. coli*); cephalothin in one case (meatal culture: *Enterobacter sp*); and gentamicin in one case (prostatic culture: *P. mirabilis*). Concordance between the bacteria that were isolated from urine and prostatic tissue was observed in only six cases, with no sensitivity to the drug utilized.

Twenty-two patients had postoperative obstruction requiring clot removal, but this did not alter the incidence of postoperative bacteriuria (p > 0.05). The same was observed for those patients who were catheterized for periods longer than three days (21 cases, 13 in the prophylaxis group and 8 in the control group).

A high prevalence of histological prostatitis (93.1%) was reported from pathological analysis with only one case of prostate adenocarcinoma.

## DISCUSSION

This study has demonstrated the irrationality of permissive antibiotic usage in transurethral resection of the prostate.

Septicemia was not observed in any case, thus differing from some studies.^6^ The heterogeneity and doubts regarding the results in the literature have arisen from the fact that most studies put patients whose preoperative urine was sterile together with those with infected urine. In relation to the latter case, the drug has a therapeutic and not a true prophylactic effect.

Clado and Halle in 1887 described "urethral fever" following catheter manipulation.^3^ Some years later, Scott demonstrated that the phenomenon was frequent but transitory, and it was possible to recover some of the bacteria from the blood sample in 62% of the cases.^2-5^ This finding led to the routine use of prophylactic antibiotic administration after any urological operation, but especially in transurethral resection of the prostate in which transected vessels are opened and become prone to direct contamination.

However, some studies have claimed that there is a disparity between the species recovered from the blood and the prostate, thereby leading to a mismatch in 27.4% of the cases.^2^ Recently, it was reported that the incidence of bacteremic episodes with clinical expression represented by chills or fever ranged from 2 to 52% of the cases.^7,8^ The source of the bacteria responsible for the chills can theoretically be the prostate gland, an ascending route outside of the catheter, the irrigating fluid or the catheter itself during and after transurethral resection of the prostate.^2,5,9^

According to most researchers, the prostate gland is the ultimate source of bacteremic episodes and is the origin of urine contamination following transurethral resection of the prostate,^10-12^ even though the bacteria recovered from the urine and glandular tissue are the same in only 36 to 50% of cases.^12^ If it is believed that transurethral resection of the prostate will lead to contamination, broadspectrum antibiotics should be preferred. If transurethral resection of the prostate is considered to be a procedure free from contamination, an appropriate choice of antibiotic must be made on the basis of the typical hospital flora that are identified, rather than on the basis of personal choice, which would mostly include drugs that are known not to reach effective tissue levels in the prostate. Therefore, the value of routine antibiotic prophylaxis can be questioned. Permissive use of antibiotics can lead to multiple antibiotic resistance, thereby favoring more difficult infections in approximately 8% of the cases.^13^

Most of the positive blood cultures usually obtained during the operation do not have any clinical significance,^4^ as demonstrated also in our data. Recently, it was reported that such culturing did not affect the incidence of fever, chills, epididymitis and urinary tract infection during the postoperative period after transurethral resection of the prostate.^8,12^

On the other hand, in patients who have a positive urine culture at the time of the operation, the occurrence of fever, bacteremic episodes and postoperative urinary tract infection can be minimized by prior treatment, as shown by other authors.^12-15^ This seems to be the rationale for the usage of antibiotic.

The ascending route has been indicated as the main reason for postoperative urinary tract infection after transurethral resection of the prostate^9^ but, far from being the sole route, this illustrates the complex and dynamic causes of urinary tract infection after transurethral resection of the prostate. Local actions can minimize local flora colonization. This can be demonstrated through the use of meatal bactericide ointment, preoperative perineal washing, auto-irrigation and early catheter removal.^16-18^

Our data revealed diminished occurrence of postoperative urinary tract infection. However, the incidence of chills, fever or hypotension, commonly seen during bacteremic episodes, and also the prevalence of microorganisms isolated from the prostatic tissue, remained unaltered with the use of antibiotics.

The limited number of cases studied does not allow definitive conclusions to be reached. This leads to a belief that the manner and criteria for antibiotic administration must be reviewed, since the practical observations in the cases without antibiotic administration showed that the clinical recovery of the patients was not compromised.

## CONCLUSIONS

Our results demonstrate that transurethral resection of the prostate can be undertaken unrestrictedly without routine antibiotic administration. However, further studies with larger samples must be undertaken so that definitive conclusions can be reached.

The protective effect of antibiotic dosage used on patients with sterile urine must be regarded critically, since such usage does not alter clinical recovery and because bacteremic episodes are multifactorial and not well understood.

Empirical usage of antibiotics does not allow matching of the bacteriological background of microorganisms isolated from different sources.
